# Dietary intake of fatty acids and risk of pancreatic cancer: Golestan cohort study

**DOI:** 10.1186/s12937-021-00723-3

**Published:** 2021-07-16

**Authors:** Neda Ghamarzad Shishavan, Sahar Masoudi, Ashraf Mohamadkhani, Sadaf G. Sepanlou, Maryam Sharafkhah, Hossein Poustchi, Mehdi Mohamadnejad, Azita Hekmatdoost, Akram Pourshams

**Affiliations:** 1 Digestive Oncology Research Center, Digestive Disease Research Institute, Shariati Hospital, Tehran University of Medical Sciences, Tehran, Iran; 2 Liver and Pancreatobiliary Diseases Research Center, Digestive Disease Research Institute, Shariati Hospital, Tehran University of Medical Sciences, Tehran, Iran; 3 Departments of Clinical Nutrition and Dietetics, Faculty of Nutrition and Food Technology, National Nutrition and Food Technology Research Institute, Shahid Beheshti University of Medical Sciences, Tehran, Iran

**Keywords:** Pancreatic cancer, Fatty acid, Dietary intake, Dietary fat, Golestan Cohort Study

## Abstract

**Background:**

As pancreatic cancer (PC) is a malignancy with poor prognosis, finding strategies for its prevention became a notable priority. Among all the factors influencing the risk of PC, dietary items especially fats are considered as the most modifiable risk factors.This study is designed to assess the associations of dietary intake of fatty acids with the risk of PC incidence.

**Methods:**

A total of 50,045 adults between 40 and 75 years old participated in this cohort study in 2004–2008 and were followed up to the present. Intakes of fatty acids was evaluated by validated food-frequency questionnaire (FFQ). Cox proportional hazards regression model was used to estimate hazard ratio (HR) with 95 % confidence interval of differing levels of dietary intakes of fatty acids for incidence of PC.

**Results:**

At the end of follow-up period, 76 cases of PC were identified and 46,904 participants without history of cancer, acute kidney disorders, fibrosis and cirrhosis were included in the study. Dietary total saturated fatty acids (SFAS) was associated with PC risk (HR = 1.05 (1.01–1.09), P_trend_=0.01), whereas dietary total monounsaturated fatty acids (MUFAS) was inversely associated with the risk of PC (HR = 0.92 (0.86–0.99), P_trend_=0.04). Dietary total polyunsaturated fatty acids (PUFAS) did show a protective but not significant association with the risk of PC (HR = 0.91(0.84-1.00), P_trend_=0.05).

**Conclusions:**

The amount of total fat intake is not a risk factor for PC in our study and focusing on the intake of specific fatty acids becomes more striking. Unsaturated fatty acids including PUFAS and especially MUFAS are considered as protective dietary factors in PC prevention. In contrast, total SFAS is positively associated with the increased risk of PC. However, very long chain and odd-chain saturated fatty acids intake may be protective against PC.

## Background

Pancreatic cancer (PC), as the seventh leading cause of cancer death among women and men, is a challenging malignancy with poor prognosis [1]. This dismal prognosis and short survival of PC patients is partly attributable to the lack of accurate screening methods or suitable tests for early detection and treatment of this cancer [2]. Thereby, primary prevention of PC is of great importance and warrants attention. Although the risk factors of PC are not known thoroughly, some predisposing factors including family history, genetic characteristics, smoking, diabetes mellitus, and obesity, are well determined. Among modifiable factors, the association of dietary elements with the risk of PC has been studied, to date [1, 3]. However, the association between certain nutrients and the risk of PC is still under investigation. Firstly, due to the inconsistent findings of previous research, and secondly, because of inadequate documents of the etiology of PC, study in this field and conception of these associations is emphatically recommended [1]. Dietary fat and its main components, fatty acids, are involved in the development of cancer [4]. The association between different types of fatty acids (FA) such as saturated fatty acids (SFAS), mono-unsaturated fatty acids (MUFAS), poly-unsaturated fatty acids (PUFAS) and trans fatty acids (TFA) with the risk of PC is yet controversial and debatable.

The general mechanism for the effects of chronic consumption of dietary fat and development of PC may be explained by the persistent secretion of cholecystokinin (CCK) hormone, which induces hyperplasia and hypertrophy in pancreatic cells [5].

Most of studies assessing the association of fat with the risk of PC have been conducted in cell line [6–9], animal models [10], interventional or case-control studies [11–14]. Furthermore, there are some cohort studies conducted worldwide on populations with dietary patterns different from those in our country [15–18].

Thus, due to the recent remarkable increase in incidence and mortality rate of PC in different parts of Iran with developing urbanization and increasing exposure to PC risk factors and unhealthy lifestyle [19], and given the lack of consistent results among Iranians, we investigated the association between dietary fat and the risk of PC in Golestan cohort study.

The results of the study will open a new window for perceiving the role of dietary fatty acids in incidence of PC, which might be worthy and promising in prevention of PC.

## Methods

### Study population

Golestan cohort study was launched in the eastern portion of the Caspian Sea littoral in northeastern Iran. The study aimed at investigating the risk factors of esophageal squamous cell carcinoma (ESCC), which were highly prevalent in that district. The cohort profile was completely explained in previous publications [20]. A total of 50,045 healthy participants were recruited in the study between 2004 and 2008. Selection of participants were on the basis of systematic cluster random sampling from the eastern three districts of Golestan Province: Gonbad (both urban and rural), Kalaleh (rural), and Aq Qala (rural). The inclusion criteria for the cohort study was the age range of 40–75 years, residing for at least ten years in that district, not having plan to emigrate in the coming five years, and not having a current or previous diagnosis of an upper gastro- intestinal (UGI) cancer.

A written informed consent, all the demographic information, a semi-quantitative food frequency questionnaire (FFQ), the anthropometric measurements, and the physical activity questionnaire were filled for all the participants at the beginning of the cohort study. Then, every participant was followed up annually by phone call and the occurrence of any disease, admission to hospital, death and its cause were inquired. Additionally, a medical team collected all the pathology reports and hospital records, and if available, tumor samples were also obtained. The study protocol was approved by the ethical review committee of the Digestive Diseases Research Institute (DDRI), affiliated to Tehran University of Medical Sciences.

### Selection of population

**Fig. 1:**
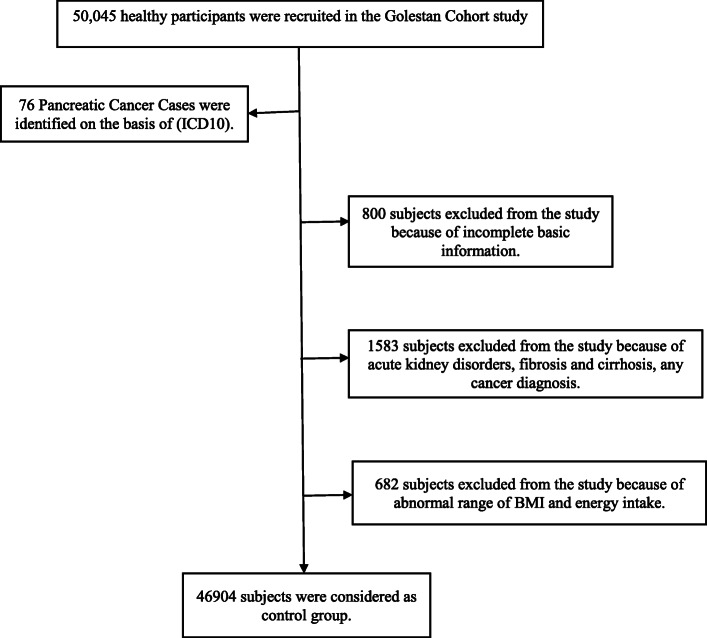
Study flow chart

In this cohort study, patients with pancreatic cancer whose cancer was confirmed during the follow up period on the basis of international classification of diseases and related health problems (ICD_10_) was assigned to the case group. The rest of the cohort population (*n* = 49,969) were considered as the control group except those who had incomplete demographic, anthropometric, or dietary data or any other basic information (*n* = 800), participants with acute kidney disorders, fibrosis and cirrhosis, any cancer diagnosis except non-melanoma skin cancer at baseline and during follow up period (*n* = 1583), the participants with body mass index (BMI) less than 15 kg/m^2^ or more than 50 kg/m^2^(*n* = 105) or those who had energy intake less than the first percentile or more than 99th percentile (*n* = 577). A consort diagram is shown in (Fig. 1).

### Dietary intake of fatty acids

A semi-quantitative food frequency questionnaire (FFQ), which its validity and reliability was confirmed in this population was used to evaluate the dietary intake of all participants at the beginning of the cohort study [20, 21]. This 116-item FFQ was filled by face to face interview by inquiring the amount of each food item by household measurements and frequency of the intake on a daily, weekly, or monthly basis during the preceding year. Then, all servings were converted into grams on the basis of the United States Department of Agriculture (USDA) table. The frequencies in every week, month, and year was converted into daily intake by dividing the numbers by 7, 30 and 365, respectively and by multiplying the frequency of each food item by the nutrient content of each food, the amount of daily intake of nutrients such as fatty acids were calculated for every participant according to the USDA composition table.

### Statistical analysis

Kolmogorov-Smirnov test was used for testing normality of variables and Independent-sample t-test, Mann-Whitney and fisher exact test were used as appropriate.

Cox proportional hazards regression model were used to estimate hazard ratio (HR) with 95 % Confidence Interval. After conducting multicollinearity test for the possible confounders (Mean VIF = 1.58), the variables including age, sex, place of residence (urban or rural), BMI, marital status, smoking, alcohol consumption, physical activity score, diabetes mellitus, total energy, and fat intake were included in the analysis as covariates.

Quantities of fatty acids were categorized into quartiles; and HR was reported for the upper three quartiles, considering the lowest quartile as the reference category. In addition to the tests for linear trend conducted based on the median value in each quartile, linear continuous changes in the intake of fats were evaluated, as well.

The restricted cubic spline (RCS) function was used to plot and investigate the possible non-linear association of each fatty acid and PC risk. In the RCS functions, we used 5 knots and set the median of the first quartile of intake as the reference point for each fatty acid.

The subgroup analysis also was carried out for smoking status, sex, BMI and ethnicity (self-report).

STATA software (version 12; STATA Corp) and R software were used for statistical analyses and *P* values (2-sided) less than 0.05 was considered as significant.

## Results

In (Table 1), some demographic characteristics of the study population in pancreatic cancer cases and controls are represented. A total of 46,980 participants (42 % men and 58 % women) were finally included in the analyses, which consists of 76 incident PC cases and 46,904 controls.

**Table 1:** Baseline characteristics of the study population (*n*= 46980)

Characteristics	Case ***N***= 76 N (%)	Control ***N***= 46904 N (%)	***P***-value
Gender			
Female	35(46.1%)	27342 (58.3%)	0.03*
Male	41(53.9%)	19562 (41.7%)	
Ethnicity			
Turkmen	63(83.9%)	34634 (73.8%)	0.07
Non-Turkmen	13(17.1%)	12270 (26.2%)	
Residence			
Rural	64(84.2%)	37013(78.9%)	0.25
Urban	12 (15.8%)	9891(21.1%)	
Education			
No formal education	57(75%)	32760 (69.8%)	0.40
Educated	19(80.3%)	14144 (30.2%)	
Marital Status			
Married	15(19.7%)	41273 (88.2%)	0.03*
Single	12(23.5%)	5546 (11.8%)	
Physical Activity			
Low	34(44.7%)	16311 (34.9%)	0.02*
Moderate	28(36.8%)	14825 (31.7%)	
Severe	14(18.4%)	15662 (33.5%)	
Wealth score			
Low	32(42.1%)	16368(34.9%)	0.420
Moderate	21(27.6%)	14599(31.1%)	
High	23(30.3%)	15937(34.0%)	
Smoker			
No	53(69.7%)	39019 (83.2%)	0.002*
Yes	23(30.3%)	7885 (16.8%)	
Opium user			
No	58(76.3%)	39173 (83.5%)	0.09
Yes	18(23.7%)	7731 (16.5%)	
Alcohol user			
No	71(93.4%)	45317 (96.6%)	0.12
Yes	5(6.6%)	1587 (3.4%)	
Family history of cancer			
No	49(64.5%)	32383(69.0%)	0.39
Yes	27(35.5%)	14521(31.0%)	
History of Diabetes			
No	73(96.1%)	43688(93.1%)	0.49
Yes	3(3.9%)	3216(6.9%)	
	Mean ± SD		*P*-value
Age (y)	58.11 ± 9.49	51.83 ± 8.80	<0.001*
Body Mass Index (BMI)	25.38 ± 5.22	26.73 ± 5.39	0.03*
	Median (percentile 25, 75)		*P*-value
Total Energy Intake	2133(1610,2618)	2123(1774,2486)	0.94
Total Fat Intake	73.41(57.92,91.32)	73.57(60.03,88.01)	0.86
Total Protein Intake	69(57,93)	73 (60,88)	0.61
Total Carbohydrate Intake	310(225,362)	303(245,358)	0.96

Data are presented as frequency (percentage) for categorical variables or Mean ± SD or Median (percentile 25, 75) for continues variables.

^*^Significant difference between two groups (*P*<0.05)

In comparison to controls, patients with PC were more likely to be smoker (*P* = 0.002) and to have less physical activity (*P* = 0.02).

On the other hand, the age distributions of cases and controls were not so similar, with an excess of younger participants in the control group in our study. The Mean ± SD age of the participants at enrollment was 51.83 ± 8.80 year in control group and 58.11 ± 9.49 year in the case group. There were no appreciable differences between two groups with respect to energy and macronutrients intakes, residence, ethnicity, education, wealth score, opium and alcohol consumption, family history of cancer and history of diabetes.

The mean lag time between age at baseline recruitment and age at PC incidence was 4.35 ± 2.15 (Minimum = 0.11, Maximum = 9.04) years.

The association between dietary intake of fatty acids and risk of PC in the continuous and quartile models are shown in (Table 2). After adjusting for all covariates including energy and total fat intake, BMI, age, sex, marital status, residence, smoking, opium consumption, diabetes, physical activity, family history of cancer, ethnicity, wealth score and education, dietary intake of total monounsaturated fatty acids (MUFAS), was inversely and total saturated fatty acids (SFAS) was directly associated with the risk of PC.

**Table 2:** HR (95% CIs) of pancreatic cancer, by quartile of each fatty acid

Fat intake	Quartiles of fat intakes				*P*-Trend	Continuous
	Q_1_	Q_2_	Q_3_	Q_4_		
**Total fat**					0.62	1.004(0.98-1.02)
Range (gday-1)	7.46-60.03	60.03-73.57	73.57-88.02	88.02-304.73		
Median (gday-1)	50.46	67.25	80.19	99.88		
Cases/controls	20/11722	18/11732	14/11730	22/11720		
Multivariate HR (95% CI)	1	0.91(0.48-1.71)	0.54(0.24-1.21)	1.07(0.44-2.59)		
**SFA**^**a**^						
4:0					0.35	0.91 (0.76-1.10)
Range (gday-1)	0.00-0.25	0.25-0.39	0.39-0.63	0.63-9.99		
Median (gday^-1^)	0.17	0.31	0.49	1.03		
Cases/controls	20/11720	13/11732	23/11724	18/11727		
Multivariate HR (95% CI)	1	0.70(0.34-1.44)	1.001(0.51-1.92)	0.73(0.36-1.46)		
6:0					0.04	0.82 (0.68-0.99) *
Range (gday-1)	0.00-0.19	0.19-0.30	0.30-0.52	0.52-9.99		
Median (gday^-1^)	0.14	0.24	0.38	3.74		
Cases/controls	23/11719	14/11727	25/11733	12/11732		
Multivariate HR (95% CI)	1	0.64(0.32-1.28)	0.81(0.43-1.53)	0.51(0.24-1.04)		
8:0					0.01	0.84(0.74-0.96) *
Range (gday-1)	0.00-0.18	0.18-0.27	0.27-0.55	0.55-10.00		
Median (gday^-1^)	0.13	0.22	0.35	5.72		
Cases/controls	23/11709	17/11736	23/11725	11/11734		
Multivariate HR (95% CI)	1	0.70(0.37-1.32)	0.65(0.34-1.24)	0.38(0.18-0.79)*		
10:0					0.55	0.93 (0.75-1.16)
Range (gday-1)	0.00-0.36	0.36-0.59	0.59-0.90	0.90-9.99		
Median (gday^-1^)	0.24	0.47	0.72	1.25		
Cases/controls	19/11722	14/11727	20/11731	21/11724		
Multivariate HR (95% CI)	1	0.82(0.40-1.68)	1.09(0.56-2.13)	0.82(0.40-1.65)		
12:0					0.37	0.89 (0.69-1.14)
Range (gday-1)	0.00-0.29	0.29-0.46	0.46-0.69	0.69-9.99		
Median (gday^-1^)	0.21	0.37	0.55	0.94		
Cases/controls	20/11722	10/11733	22/11724	22/11725		
Multivariate HR (95% CI)	1	0.55(0.25-1.19)	1.13(0.58-2.18)	0.81(0.40-1.64)		
14:0					0.78	1.04 (0.77-1.41)
Range (gday-1)	0.10-0.85	0.85-1.32	1.32-1.93	1.93-9.91		
Median (gday^-1^)	0.57	1.08	1.59	2.47		
Cases/controls	17/11726	13/11732	19/11726	25/11720		
Multivariate HR (95% CI)	1	0.88(0.41-1.85)	1.17(0.57-2.43)	1.06(0.47-2.37)		
16:0					0.30	0.91 (0.77-1.08)
Range (gday-1)	1.09-8.20	8.20-10.26	10.26-12.67	12.67-54.39		
Median (gday^-1^)	6.84	9.27	11.34	14.73		
Cases/controls	19/11725	13/11732	19/11726	23/11721		
Multivariate HR (95% CI)	1	0.53(0.24-1.18)	0.72(0.32-1.63)	0.68(0.21-2.12)		
18:0					0.01	1.03(1.01-1.07)
Range (gday-1)	0.61-18.17	18.17-24.65	24.65-30.90	30.90-234.84		
Median (gday^-1^)	12.58	21.68	27.68	35.78		
Cases/controls	16/11728	18/11726	14/11732	26/11718		
Multivariate HR (95% CI)	1	1.21(0.58-2.54)	1.24(0.54-2.87)	2.57(1.03-6.41)*		
20:0					0.33	-
Range (gday-1)	0.00-0.09	0.09-0.14	0.14-0.18	0.18-1.08		
Median (gday^-1^)	0.06	0.12	0.16	0.21		
Cases/controls	21/11722	10/11712	20/11738	23/11732		
Multivariate HR (95% CI)	1	0.53(0.24-1.18)	0.97(0.47-2.01)	1.25(0.53-2.92)		
22:0					0.01	-
Range (gday-1)	0.00-0.08	0.08-0.11	0.11-0.15	0.15-1.08		
Median (gday^-1^)	0.05	0.10	0.13	0.17		
Cases/controls	18/11719	13/11726	20/11738	23/11721		
Multivariate HR (95% CI)	1	0.74(0.34-1.59)	1.47(0.70-3.08)	1.80(0.76-4.28)		
24:0					<0.001	0.69(0.60-0.80) *
Range (gday-1)	0.00-2.60	2.60-3.69	3.69-4.82	4.82-9.99		
Median (gday^-1^)	1.90	3.17	4.20	5.80		
Cases/controls	36/11704	8/11737	13/11731	17/11726		
Multivariate HR (95% CI)	1	0.24(0.11-0.52) *	0.36(0.18-0.69)*	0.42(0.22-0.81)*		
Odd carbon Fatty acids (13:0,15:0,17:0)					<0.001	0.76(0.67-0.85) *
Range (gday-1)	0.00-1.65	1.65-3.07	3.07-5.85	5.85-10.00		
Median (gday^-1^)	1.24	2.22	4.29	7.78		
Cases/controls	32/11713	19/11725	15/11729	8/11737		
Multivariate HR (95% CI)	1	0.72(0.40-1.28)	0.49(0.26-0.91)*	0.27(0.12-0.61)*		
Long Chain fatty acids (>20C)					<0.001	0.71(0.61-0.82) *
Range (gday-1)	0.00-2.79	2.79-3.95	3.95-5.15	5.15-10.80		
Median (gday^-1^)	2.05	3.41	4.50	6.18		
Cases/controls	36/11709	8/11733	13/11730	17/11726		
Multivariate HR (95% CI)	1	0.24(0.11-0.52) *	0.36(0.18-0.69)*	0.42(0.22-0.81)*		
**Total SFA**					0.01	1.05 (1.01-1.09) *
Range (gday-1)	1.78-30.28	30.28-39.22	39.22-47.94	47.94-270.28		
Median (gday^-1^)	23.72	35.12	43.29	54.77		
Cases/controls	19/11725	18/11725	11/11736	26/11718		
Multivariate HR (95% CI)	1	0.95(0.46-1.96)	0.73(0.29-1.82)	1.63(0.56-4.75)		
**MUFA**^**a**^**:**						
16:1 *undifferentiated*					0.06	0.50(0.24-1.03)
Range (gday-1)	0.026-0.54	0.54-0.76	0.76-1.03	1.03-7.53		
Median (gday^-1^)	0.41	0.65	0.88	1.28		
Cases/controls	23/11718	14/11731	15/11730	22/11725		
Multivariate HR (95% CI)	1	0.54(0.27-1.08)	0.50(0.25-1.03)	0.42(0.20-0.90)*		
18:1 *undifferentiated*					0.04	0.92(0.85-0.99) *
Range (gday-1)	1.38-13.57	13.57-17.16	17.16-21.33	21.33-80.80		
Median (gday^-1^)	11.22	15.39	19.04	24.89		
Cases/controls	19/11725	20/11724	19/11727	16/11728		
Multivariate HR (95% CI)	1	0.65(0.32-1.32)	0.54(0.23-1.25)	0.34(0.11-1.05)		
20:1					0.91	1.00(0.91-1.10)
Range (gday-1)	0.00-0.16	0.16-.020	0.20-0.29	0.29-9.99		
Median (gday^-1^)	0.12	0.17	0.23	4.14		
Cases/controls	16/11566	17/11575	21/11573	20/11580		
Multivariate HR (95% CI)	1	0.84(0.41-1.71)	1.39(0.69-2.79)	1.12(0.57-2.20)		
**Total MUFA**					0.036	0.92(0.86-0.99) *
Range (gday-1)	1.58-14.59	14.59-18.46	18.46-22.94	22.94-89.58		
Median (gday^-1^)	12.09	16.55	20.47	26.74		
Cases/controls	19/11725	20/11724	19/11726	16/11729		
Multivariate HR (95% CI)	1	0.68(0.34-1.36)	0.50(0.22-1.16)	0.32(0.10.0.99)*		
**PUFA n-6:**						
20:2n-6					0.81	-
Range (gday-1)	0.00-0.003	0.003-0.006	0.006-0.009	0.009-0.096		
Median (gday^-1^)	0.00	0.00	0.01	0.01		
Cases/controls	17/11214	19/12240	20/11671	18/11779		
Multivariate HR (95% CI)	1	1.67(0.84-3.32)	1.90(0.95-3.80)	1.60(0.76-3.39)		
18:2n-6					0.78	0.97(0.83-1.14)
Range (gday-1)	0.00-0.79	0.79-1.67	1.67-2.66	2.66-37.93		
Median (gday^-1^)	0.18	1.23	2.12	3.61		
Cases/controls	22/11685	18/11762	19/11729	15/11728		
Multivariate HR (95% CI)	1	1.24(0.65-2.37)	1.26(0.66-2.41)	0.95(0.46-1.96)		
18:2 n-6 (CLA)					0.39	-
Range (gday-1)	0.00-0.00	0.00-0.00	0.00-0.00	0.00-0.20		
Median (gday^-1^)	0.00	0.00	0.00	0.01		
Cases/controls	40/22510	1/928	14/10440	19/13026		
Multivariate HR (95% CI)	1	0.66(0.09-4.83)	0.94(0.50-1.75)	0.95(0.52-1.70)		
**18:2** Undifferentiated					0.08	0.92(0.84-1.01)
Range (gday-1)	0.47-5.71	5.71-7.41	7.41-9.76	9.76-123.04		
Median (gday^-1^)	4.68	6.56	8.39	12.56		
Cases/controls	23/11721	22/11723	13/11732	16/11728		
Multivariate HR (95% CI)	1	0.61(0.32-1.17)	0.40(0.18-0.88)*	0.47(0.19-1.15)		
**18:3** Undifferentiated					0.14	0.31(0.06-1.48)
Range (gday-1)	0.09-0.56	0.56-0.72	0.72-0.91	0.91-6.08		
Median (gday^-1^)	0.46	0.64	0.80	1.06		
Cases/controls	20/11715	18/11734	16/11722	20/11733		
Multivariate HR (95% CI)	1	0.82(0.40-1.69)	0.63(0.27-1.47)	0.68(0.24-1.89)		
**20:4** Undifferentiated					0.73	0.98(0.88-1.08)
Range (gday-1)	0.00-0.17	0.17-0.25	0.25-0.44	0.44-9.99		
Median (gday^-1^)	0.13	0.21	0.31	4.77		
Cases/controls	23/11464	20/11486	17/11482	13/11486		
Multivariate HR (95% CI)	1	0.87(0.47-1.59)	0.66(0.34-1.27)	0.38(0.18-0.78)*		
**PUFA n-3:**						
20:5n-3(EPA)					0.74	-
Range (gday-1)	0.00-0.01	0.01-0.02	0.02-0.04	0.04-1.59		
Median (gday^-1^)	0.00	0.01	0.02	0.06		
Cases/controls	19/11686	21/11696	17/11791	17/11731		
Multivariate HR (95% CI)	1	1.19(0.63-2.23)	0.95(0.48-1.90)	0.84(0.40-1.75)		
22:5n-3(DPA)					0.12	-
Range (gday-1)	0.00-0.01	0.01-0.03	0.03-0.05	0.05-0.55		
Median (gday^-1^)	0.01	0.02	0.04	0.06		
Cases/controls	20/11689	19/11744	17/11699	18/11772		
Multivariate HR (95% CI)	1	1.00(0.53-1.88)	0.95(0.49-1.84)	0.66(0.33-1.35)		
18:3n-3(ALA)					0.45	1.88(0.35-9.90)
Range (gday-1)	0.00-0.02	0.02-0.03	0.03-0.05	0.05-4.71		
Median (gday^-1^)	0.02	0.03	0.04	0.08		
Cases/controls	19/11712	14/11692	19/11764	22/11736		
Multivariate HR (95% CI)	1	0.76(0.36-1.59)	1.18(0.57-2.41)	1.64(0.77-3.48)		
22:6n-3(DHA)					0.28	0.09(0.001-7.25)
Range (gday-1)	0.00-0.03	0.03-0.06	0.06-0.09	0.09-1.62		
Median (gday^-1^)	0.02	0.04	0.07	0.13		
Cases/controls	20/11688	20/11738	15/11750	19/11728		
Multivariate HR (95% CI)	1	1.05(0.56-1.97)	0.81(0.41-1.63)	0.69(0.34-1.39)		
**Total PUFA**^**a**^					0.05	0.91(0.84-1.00)
Range (gday-1)	0.66-6.67	6.67-8.62	8.62-11.29	11.29-125.45		
Median (gday^-1^)	5.49	7.64	9.74	14.38		
Cases/controls	23/11721	22/11722	11/11734	18/11727		
Multivariate HR (95% CI)	1	0.72(0.38-1.35)	0.24(0.10-0.57) *	0.48(0.19-1.17)		
**Total n-3**^**a**^					0.52	0.52(0.07-3.76)
Range (gday-1)	0.00-0.10	0.10-0.16	0.16-0.24	0.24-4.90		
Median (gday^-1^)	0.07	0.13	0.19	0.33		
Cases/controls	17/11716	23/11717	17/11738	17/11733		
Multivariate HR (95% CI)	1	1.42(0.75-2.69)	1.11(0.54-2.25)	0.70(0.32-1.53)		
**Total n-6**^**a**^					0.77	0.97(0.83-1.14)
Range (gday-1)	0.00-0.79	0.79-1.68	1.68-2.68	2.68-37.93		
Median (gday^-1^)	0.19	1.24	2.12	3.61		
Cases/controls	22/11721	18/11727	19/11722	15/11734		
Multivariate HR (95% CI)	1	1.24(0.65-2.39)	1.26(0.66-2.42)	0.95(0.46-1.96)		
**Total trans**					0.05	1.09(0.99-1.20)
Range (gday-1)	0.00-4.60	4.60-6.56	6.56-8.35	8.35-70.12		
Median (gday^-1^)	3.01	5.67	7.48	9.66		
Cases/controls	20/11725	16/11727	15/11731	25/11721		
Multivariate HR (95% CI)	1	0.89(0.43-1.83)	1.10(0.51-2.39)	1.92(0.84-4.41)		

HR (95%CI) for Cox proportional hazards regression model adjusted for energy and total fat intake, BMI, age, gender, marital status, residence, smoking, diabetes, physical activity, opium consumption, family history of cancer, ethnicity, wealth score and education

^a^*SFA* Saturated fatty acids, *MUFA* Mono unsaturated fatty acids, *PUFA* Poly unsaturated fatty acids, *TFA* Trans fatty acids, n-3: Omega-3 fatty acids, n-6: Omega-6 fatty acids

^*^Significant difference between quartiles (*P*<0.05)

Although total intake of SFAS was associated with increased risk of PC (HR = 1.05 (1.01–1.09), P_trend_=0.01), some short chain-SFAS including 6:0 and 8:0, and some long chain-SFAS such as fatty acids more than 20 carbons were inversely associated with the risk of PC (Table 2).

Furthermore, stearic acid (18:0) intake was associated with the increased risk of PC (HR = 1.03(1.01–1.07), P_trend_=0.01). On the other hand, PUFAS intake was associated with lower risk of PC, even if this association was not significant (HR = 0.91(0.84-1.00), P_trend_=0.05).

Moreover, further analysis was conducted to assess the association of fatty acids depending on food source and PC risk. Fatty acids from red meat, chicken, fish, dairy and oil, separately, were calculated and none of them showed significant association with the risk of PC in both continuous and quartile models [data not shown].

Upon stratification by sex, BMI, and ethnicity, we found that the potential predisposing effects of total SFAS and the protective effect of total MUFAS on PC risk became significant in men, non-smokers and participants with BMI < 30. Ethnicity stratification (Turkmen and non-Turkmen groups) did not affect the association between PC risk and fatty acid groups except for total SFAS which was a risk factor especially in non-Turkmen compared Turkmen group. The stratification did not reveal any significant association between other fatty acid groups including total PUFAS and trans- fatty acid with PC risk.

**Fig. 2:**
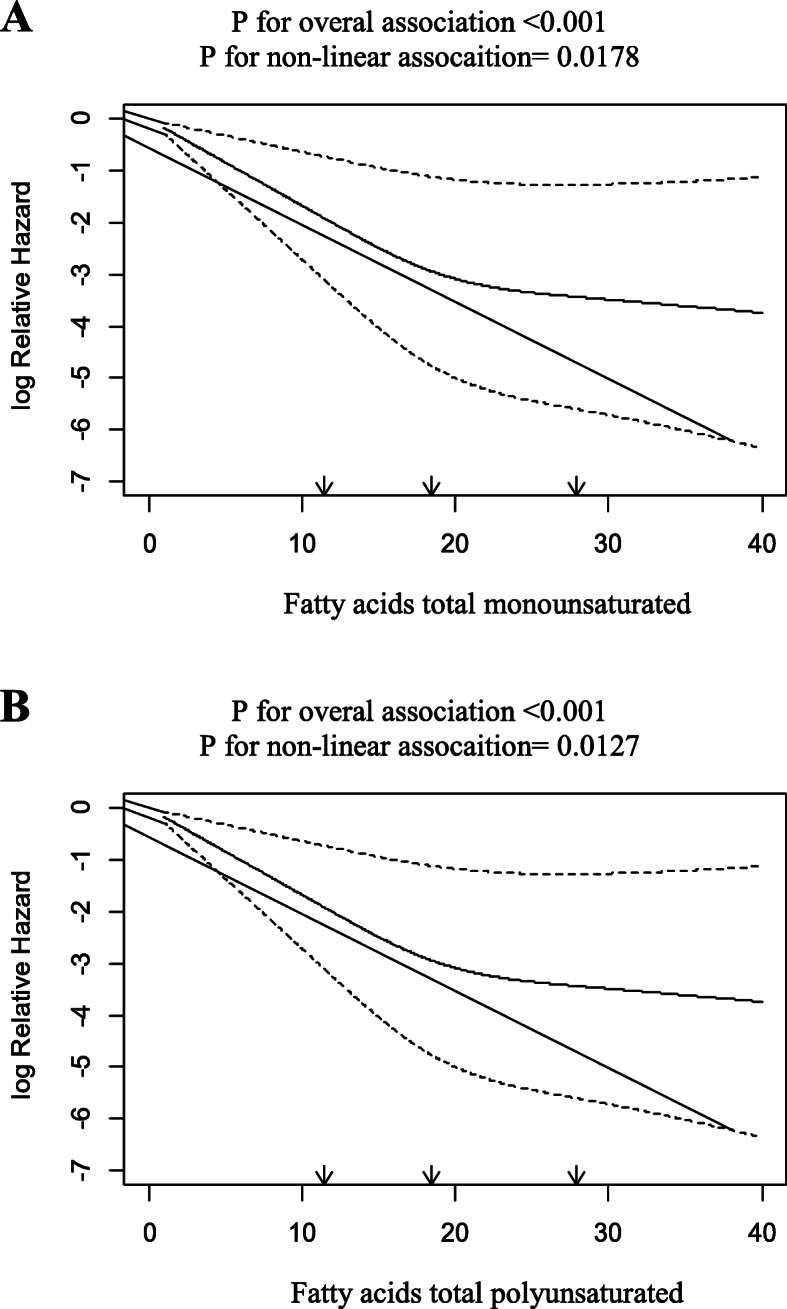
Associations between intakes of total MUFA and total PUFA with the risk of PC. Curved solid lines represent adjusted HR and dashed lines indicate their 95 % CIs based on restricted cubic splines

In order to assess the non-linear association between different classes of fatty acids and PC risk, RCS model was conducted for all the total fatty acid groups including total SFAS, MUFAS, PUFAS, Trans, Omega-3 fatty acids, Omega-6 fatty acids, odd-chain saturated fatty acids and very long chain fatty acids adjusting for some variables such as age, sex, energy and total fat intake, BMI, marital status, residence, smoking, opium consumption, diabetes, physical activity, family history of cancer, ethnicity, wealth score and education. Although there was a nonlinear association between total MUFAS and PUFAS with the risk of PC, no clear trend of intake was observed (Fig. 2).

## Discussion

In this cohort study, we found that people who consume more SFAS are more likely to develop PC. Furthermore, our results showed that total MUFAS consumption is associated with decreased risk of PC. We did not observe any significant association of total PUFAS and total TFA intake with PC risk.

### Total fat intake

There was no association between total fat and risk of PC in our study, which concurred with the previous documents [12, 22]. However, some other researchers reported a different finding from ours, which found a negative [23, 24], or positive [11, 13, 15, 16] relation between total fat intake and PC risk.

Our study did not show any significant association between total fat from some food groups (red meat, chicken, dairy and fish) and PC risk (data not shown).

The risk of pancreatic tumorigenesis in rats receiving high-fat diet was higher than those receiving low-fat diet with the same calorie intake [25].Therefore, this implies that dietary fat intake is more important than calorie intake in the development and progression of PC [25, 26]. After food ingestion and fat hydrolysis, fatty acids are transported through chylomicron to the duodenum and induce cholecystokinin (CCK) secretion. In the cases of chronic fat intake, long-lasting CCK secretion and consequently chronic cholecystokininemia, stimulate pancreatic enzymes secretion, hyperplasia and hypertrophy of pancreas [5]. This mechanism indicates a high risk of PC in case of high and constant fat intake. Thus, the null result in our study might be attributed to optimum intake of fat in the participants of the study. WHO-2018 guideline on healthy diet, emphasizes that less than 30 % of total calorie intake should be obtained from fat groups [27]. According to our findings, we found that total fat intake does not exceed 30 % of total calorie intake in each quartile.

### Saturated fatty acids

In the present study, not only some short chain SFAS including Caproic acid (6:0) and Caprylic acid (8:0), but also very long chain saturated fatty acids (VLSFAS) (≥ 20 C) were associated with decreased risk of PC. However, among dietary very long chain fatty acids including Arachidic acid (20:0) and Behenic acid (22:0), only Lignoceric acid (24:0), with high dietary intake among other VLSFAS class, reduced PC risk by approximately 30 % (in the continuous model).The protective effects of this class of fatty acids were previously reported for the risk of metabolic syndrome, diabetes and cardiovascular diseases [28, 29]. In addition to the confirmed role of obesity and diabetes in developing PC [30], findings of a meta-analysis of 4 cohort studies investigating the relation of metabolic syndrome (Mets) and pancreatic cancer risk, demonstrated a high risk of PC in individuals with MetS [RR = 1.55 (95 % CI, 1.19–2.01)] [31].

Even though the exact mechanism underlying the health effects of VLSFAS needs to be determined, a family of waxy lipid molecules called ceramides and sphingomyelin, which are mainly composed of VLSFAS, are associated with induction of apoptosis, insulin sensitivity and anti-inflammatory response through signaling pathways [28].

Overall, we found that people who consume high amount of dietary total SFAS are more likely to develop PC; this might be the result of high percentage of stearic acid (18:0) intake which accounted for about 50 % of the total SFAS intake in our study population, and is significantly associated with the increased risk of PC.

We also evaluated the intake of odd-chain saturated fatty acids including 13:0, 15:0 and 17:0. These fatty acids constitute a small amount of SFAS intake in the diet, and are mainly found in dairy products. These fatty acids were associated with decreased risk of PC, which is completely in line with previous research indicating their protective property against diabetes mellitus [32, 33]. Forouhi N.G, et al., asserted that increase in even-chain SFAS in result of de-novo lipogenesis not only contributes to some metabolic pathways, hepatic steatosis and finally type 2 diabetes mellitus, but also causes inflammatory cytokines activation directly. However, there was a significant inverse association between SFA derived dairy products especially odd-chain SFAS with the risk of diabetes [33] which is one of the determined risk factors for PC.

Some previous studies indicated that SFAS intake is not associated with the risk of PC [12, 16, 22]. Yet, there is evidence showing high intake of SFAS is a risk factor for PC [11, 14, 15, 34]. Overall, it is suggested that saturated fat intake influences the risk of cancer through several mechanisms including insulin resistance, DNA damage and enzyme secretion, which all lead to carcinogenesis [35–39].

Current study showed a positive slight association between dietary total SFAS and PC risk, which could plausibly account for higher mean intake of total SFAS in this population than the WHO-2018 guideline on healthy diet (< 10 % of calorie intake) [27]. Previous studies also reported increased risk of PC with SFAS, which was conducted in only French Canadians or male smokers who have special dietary pattern with high SFAS intake. It might be concluded that this association was observed only in the population with high SFAS intake [15]. We also found that dietary SFAS was associated with the increased risk of PC especially in men and the participants without other risk factors such smoking and obesity (BMI < 30).

### Unsaturated fatty acids

#### PUFAS

Our findings showed that intake of PUFAS was not significantly associated with the risk of PC, which is in agreement with some observational studies [11]. While, in other studies, a protective [13, 14, 16] or even a positive association [11, 12, 15] was found. In the quartile model, intake of PUFAS in the quartiles 3 vs. quartile 1, was significantly associated with decreased risk of PC by 76 %.

Although there is not yet any explanation for the mechanisms of dietary total PUFAS in cancer, it is thought that the effects of two classes of PUFAS especially omega-3 and omega-6 fatty acids, as essential fatty acids, should be separately considered in PC risk.

The protective effects of omega-3 fatty acids are definitely confirmed in prevention of many diseases and on the basis of previous findings, omega-3 fatty acids significantly decreased risk of PC through the anti-inflammatory and immunoregulatory properties, insulin sensitivity improvement and the direct effect on DNA and apoptosis pathways [34, 40].

Previous research showed an inverse association between intake of linoleic acid (LA, an omega-6 fatty acid,18:2n-6) and the risk of PC, as well [14]. Moreover, another study reported a great resistance against injury by bile in rats fed a diet rich in linoleic acid [41]. On the contrary, some studies indicated that linoleic acid increased the risk of PC, significantly [34]. Even though the daily intake of these fatty acids is necessary, it seems that the ratio of omega-6 to omega-3 fatty acids in the diet is substantial in determination of the risk of many diseases. These fatty acids compete with each other in biosynthesis of eicosanoids like prostaglandins, which are involved in tumor promotion [14, 22]. Thus, the ratio in prevention of many disorders deserves more attention. However, our findings did not show any significant association between the ratio of omega-6 to omega-3 fatty acids, total omega-3, total omega-6 and the risk of PC.

#### MUFAS

We found a significant inverse association between intake of MUFAS and risk of PC. In the present study 68 % reduction and in the study by Nkondjock A, et al., 28 % reduction [39], in the highest quartile of MUFAS intake compared to the lowest was observed.

Additionally, in the current study, the association became stronger and more remarkable when considered only in men, non-smokers and non-obese participants (BMI < 30) [HR = 0.87(0.78–0.96), HR = 0.89(0.82–0.97) and HR = 0.89(0.82–0.96); for all of them P_trend_<0.01, respectively]. This is in agreement of the findings between plasma levels of MUFA and PC risk in this population [42].

Another case-control study indicated the protective effects of MUFAS with 90 % reduction of PC risk [43]. However, no significant association was found between MUFAS intake and the risk of PC in some other studies [12, 22].

Gong Z, et al., found that certain MUFAS including Palmitoleic acid (16:1n-9) and Oleic acids (18:1n-9) may increase the risk of PC; on the other hand, a member of MUFAS, Gadoleic acid (20:1n-11) intake, was associated with the decreased risk of PC. The authors justified this finding with different food sources of MUFAS in the diet. Even though, vegetable oils and nuts are rich in MUFAS, they showed that the main sources of MUFAS in Europe, and U.S. diet are animal products, so because of other components of animal foods, these effects cannot be exactly attributed to the MUFAS content [34].

A pooled analysis of the Netherlands Cohort Study and the Dutch cohort of the European Prospective Investigation into Cancer and Nutrition, reported no significant positive association between Mediterranean diet and risk of PC [44]. Schulpen M, et al., asserted that their findings are more accurate than others owing to the conduction of the study in a homogene group of patients (only microscopically confirmed pancreatic cancer cases) [44].

However, Banim PJ, et al., in the European Prospective Investigation of Cancer-Norfolk Study (EPIC-Norfolk), reported a significant inverse association between dietary intake of Oleic acid and the risk of PC particularly in those with high BMI (BMI > 25) [45]. They emphasized that oleic acid significantly increased insulin sensitivity which was measured by serum HbA_1_c. As previously confirmed, insulin not only influences malignant cancer cells promotion directly via some signaling pathways, but also induces carcinogenic effects indirectly through production of some inflammatory cytokines such as interleukin-6 (IL-6) and C-reactive protein (CRP) [45, 46]. We also did demonstrate this protective effect for dietary total MUFAS and PC risk. These discrepant results might be explained by different proportion of certain fatty acids in the diet or diverse percentage of total energy intake from fat [23].

On stratification analysis by sex, BMI, and smoking, we found out that all associations between SFAS and MUFAS with the risk of PC became stronger in participants who did not smoke, in men, and in non-obese participants (BMI < 30). Since it seems that the main reason for this finding lies in the fact that smoking and obesity are considered as confirmed risk factors for PC, the association between different types of fatty acids and PC become negligible in the at risk group.

Our study has certain noteworthy strengths. Since all dietary intake data were collected years before the diagnosis of cancer in our study, the data was absolutely not affected by the other factors and the biased risk which may probably occur in the case-control studies. In addition, due to the large sample size (~ 50,000 participants), the study has high power to detect any differences in determination of risk factors. Also, use of the valid food frequency questionnaire with 116-items provided a comprehensive evaluation of dietary intake of fatty acids. Overall, these are the strengths of our study which increase the accuracy and precision of the data.

## Conclusions

In conclusion, we found an inverse association between intake of MUFAS (and PUFAS in a quartile model, quartile 3 vs. the lowest quartile, not in higher intakes) with the risk of PC. However, we observed that SFAS was a prominent risk factor for PC. Trans-fatty acids (TFA) also were non-significantly associated with the increased risk of PC. Reducing intake of SFAS food sources like butter, coconut oil and palm kernel oil and replacing them with the healthier food items like MUFAS sources such as olive oil and canola oil can substantially reduce the risk of PC.

All authors read and approved the final manuscript and they declare that none of them are employed by a government agency or are not submitting this manuscript as an official representative or on behalf of the government.

## Data Availability

Data cannot be made freely available as they are subject to secrecy in accordance with the Golestan Cohort Study protocol, but are available from the corresponding author on reasonable request (akrampourshams@gmail.com).

## Abbreviations

PC: Pancreatic Cancer
HR: Hazard ratio
FAs: Fatty acids
SFA: Saturated fatty acids
MUFA: Mono-unsaturated fatty acids
PUFA: Poly-unsaturated fatty acids
TFA: Trans fatty acids
CCK: Cholecystokinin
ESCC: Esophageal squamous cell carcinoma
UGI: Upper gastro- intestinal
FFQ: Food-frequency questionnaire
DDRI: Digestive Diseases Research Institute
ICD10: International classification of diseases
USDA: The United States Department of Agriculture
BMI: Body mass index
RCS: Restricted cubic spline
VLSFAS: Very long chain saturated fatty acids
LA: Linoleic acid
